# Sensitive and Specific Biomimetic Lipid Coated Microfluidics to Isolate Viable Circulating Tumor Cells and Microemboli for Cancer Detection

**DOI:** 10.1371/journal.pone.0149633

**Published:** 2016-03-03

**Authors:** Jia-Yang Chen, Wen-Sy Tsai, Hung-Jen Shao, Jen-Chia Wu, Jr-Ming Lai, Si-Hong Lu, Tsung-Fu Hung, Chih-Tsung Yang, Liang-Chun Wu, Jinn-Shiun Chen, Wen-Hwa Lee, Ying-Chih Chang

**Affiliations:** 1 Genomics Research Center, Academia Sinica, Nankang, Taipei, Taiwan; 2 Division of Colon and Rectal Surgery, Chang Gung Memorial Hospital, Kueishan, Taoyuan, Linkou, Taiwan; 3 Graduate Institute of Clinical Medical Science, Chang Gung University, Taoyuan, Taiwan; 4 Graduate Institute of Life Sciences, National Defense Medical Center, Taipei, Taiwan; 5 Graduate Institute of Clinical Medical Science, China Medical University, Taichung, Taiwan; The Ohio State University, UNITED STATES

## Abstract

Here we presented a simple and effective membrane mimetic microfluidic device with antibody conjugated supported lipid bilayer (SLB) “smart coating” to capture viable circulating tumor cells (CTCs) and circulating tumor microemboli (CTM) directly from whole blood of all stage clinical cancer patients. The non-covalently bound SLB was able to promote dynamic clustering of lipid-tethered antibodies to CTC antigens and minimized non-specific blood cells retention through its non-fouling nature. A gentle flow further flushed away loosely-bound blood cells to achieve high purity of CTCs, and a stream of air foam injected disintegrate the SLB assemblies to release intact and viable CTCs from the chip. Human blood spiked cancer cell line test showed the ~95% overall efficiency to recover both CTCs and CTMs. Live/dead assay showed that at least 86% of recovered cells maintain viability. By using 2 mL of peripheral blood, the CTCs and CTMs counts of 63 healthy and colorectal cancer donors were positively correlated with the cancer progression. In summary, a simple and effective strategy utilizing biomimetic principle was developed to retrieve viable CTCs for enumeration, molecular analysis, as well as *ex vivo* culture over weeks. Due to the high sensitivity and specificity, it is the first time to show the high detection rates and quantity of CTCs in non-metastatic cancer patients. This work offers the values in both early cancer detection and prognosis of CTC and provides an accurate non-invasive strategy for routine clinical investigation on CTCs.

## Introduction

Metastasis is the main cause of recurrence and mortality in patients with solid-tumors worldwide. It is believed that once the primary tumor is established, additional mutations and the microenvironment interactions of the cancer cells will promote dissemination for cancer metastasis. The epithelial-mesenchymal transition (EMT) has been implicated as being responsible for the shedding of tumor cells from adherent epithelial cells in preclinical models [1]. Intravasation of epithelial origin primary cancer cells will allow the cancer cells circulate into blood stream as circulating tumor cells (CTCs) through migration/invasion progression. The disseminated CTCs may thus travel some distance and colonize at secondary sites for metastatic tumor establishment. But the mechanism of cancer metastasis is still obscure and perception of cancer dissemination as CTCs remains a challenge. CTCs are evasive to detection because of the extremely rare population in the circulation of the cancer patients. It could be as few as only 1~1000s CTCs out of billions of blood cells in symptomatic cancer patients. Despite its rare population, the quantity of CTCs in the blood has shown to correlate with the poor prognosis of the metastatic cancer patients [2], and outcomes of chemotherapy in breast, prostate, and melanoma cancer patients [1,3,4]. These studies indicated that monitoring of CTC counts may be useful for early detection and efficacy monitoring during treatment.

Recently, emerging evidence showed that the presence of circulating tumor microemboli (CTM) is strongly associates with distant metastasis. In comparison with the presence of single CTCs alone, the presence of CTMs correlated well with the poor prognosis in metastatic breast, prostate, and small cell lung cancers [5,6]. It has been proposed that cell aggregates, such as CTMs, provide a cell-cell adhesion advantage against shear stress in the blood stream and activate signaling for anti-apoptosis and protection from anoikis [5]. Evidence of collective movement in primary tumor cells through a β1-integrin-dependent manner provides an opportunity of shedding CTMs into the blood stream [7]. Abandonment of plakoglobin-mediated cell-cell interaction results in a decrease of CTMs in the blood stream and correlates with better prognosis [6]. Despite the significant role of CTCs, the role of CTMs and the interactions between CTCs and the microenvironment during cancer progression is still unclear. Enumeration and characterization of the identified/purified CTCs from cancer patients will uncover the character of CTCs/CTMs in cancer progression. Establishment of a CTCs capture system that permits high sensitivity, specificity, and viability for both CTCs and CTMs will provide great benefit for the diagnosis and treatment of clinical cancer patients.

Various technologies have disclosed CTCs enrichment and identification based on different principles, including immuno-magnetic isolation [8–14], cell-size based filtration [15,16], antibody-functionalized microfluidic devices [17–21], fiber-optic array scanning technology [22], dielectrophoresis, passive cell sorting [23], negative selection [24,25], ensemble-decision aliquot ranking [26], nano-roughened adhesion surface [27], thermo-responsive polymer coating [28], or combinations of the above [29,30]. Some of these technologies showed better sensitivity than others, including anecdotal studies in non-metastatic diseases [31]. However, hardly any has proven clinical utility in routine detection of CTCs for all-stages of cancers as this requires a highly sensitive and specific platform to isolate and preserve patient-derived CTCs for further investigation.

Of all the affinity-based methods, microfluidic devices are emerging as promising tools to efficiently isolate rare cells, including CTCs and stem cells [18,32–34]. The main challenge for this promising platform is to release and recover the isolated target cells with biological activity [35]. By using enzymatic digestion, the release of captured cells may disrupt the cell membrane, degrade surface markers, and alter both phenotypic and functional information of the CTCs, thus limiting the downstream analyses of cells [34,36]. In addition, although the flow-induced cell detachment shows good recovery efficiency from 60% to 90% [37–39], the 50 to 200 dynes/cm^2^ high shear stress is necessary to detach cells by breaking the antibody-cell surface antigen bonds [40]. Such a strong force is known to alter gene expression and possibly lead to phenotypical changes and cell death [32,41,42]. Bubble-induced detachment of affinity-adsorbed blood cells, which leverage the air-liquid interfacial tension exerted upon the adhered cell to uproot it from the surface with 90 to 100% release efficiency in microstructure-free straight channels. However, low cell viability and inconsistent recovery rates have not been properly addressed [43–45].

Among these studies, however, surface effects have been overlooked. Conceptually, a “non-fouling” surface, i.e., a surface with minimal physical interaction with cells, could reduce the shear stress required to flush away non-specifically adhered cells and provide an opportunity to reduce the force necessary for cell desorption. Furthermore, a coating designed with weak bonding to the surface such as physisorption could provide non-disrupting cleavage points (the weakest links) when exerting interfacial tension (such as air bubbles). The ability to interrupt the surface coating prevents direct cleavage of cell membrane receptor bonds, which most often result in cell damage. In this communication, we describe a smart coating in a microfluidic platform that provides “non-fouling” property to increase rejection of unwanted materials in the blood and the “weakest links” for cell release. Here we report the supported lipid bilayer (SLB) coated microfluidic system, CMx platform (“Cells captured in Maximum”), and prove to be highly sensitive and specific for capturing both single CTCs and CTMs and feature with easy-to-release platform for acquiring viable CTCs; all requires only 2 mL of whole blood. A clinical study involving enumeration of both CTCs and CTMs based on 9 healthy donors confirmed by colonoscopy and 54 patients with stages I to IV of colorectal cancer (CRC) further confirmed the superior sensitivity and clinical utility of the fluidic lipid coated platform. Concomitant patient-derived CTC culture and mutation analysis, further improved the feasibility and utility for both basic research and clinical treatment of the CMx platform.

## Materials and Methods

### Microfluidic Chip Preparation and Surface Coating

The fabrication of custom antibody-conjugated SLB (Ab-SLB) coated microfluidic chips, the CMx platform, is described as follows: A commercial CO_2_ laser scriber (Epilog Helix 24, Golden, CO) is used to create micropatterns on the poly(methyl methacrylate) (PMMA) slide (size = 76 mm x 25.4 mm, thickness = 1.5 mm). The laser is also used to engrave a 63 μm-thick double-side adhesive tape (8018PT; 3M Corp, Maplewood, MN) to carve out the borders surrounding the microfluidic patterns on the PMMA. The microtrenched patterns and microstructures on the PMMA slide were drawn using CorelDraw (Corel, Ottawa, Canada) and then transferred to the laser scriber for direct machining on the substrate. In this study, six types of chips were prepared. The engraved chips were bonded with the plasma treated glass slide by placing the carved out 3M adhesive tape (sandwiched) between the top (PMMA slide) and a glass slide on the bottom to form sealed channels. The preparation of lipid vesicles, biotinylation of antibody of EpCAM, EpAb4-1, and the sequential preparation of anti-EpCAM-supported lipid bilayer coating in the microfluidic chip were described previously [46]. In brief, the inner walls of the microfluidic channels were treated with lipid vesicles consisting of 1-palmitoyl-2-oleoyl-*sn*-glycero-3-phosphocholine (POPC) and 1,2-dipalmitoyl-*sn*-glycero-3-phosphoethanolamine-N-cap-biotinyl (b-PE) in molar ratios of 95/5 to form SLB. For fluorescence conjugated SLB (Texas Red-SLB), the microfluidic channels were coated by lipid vesicles consisting of POPC, b-PE, and Texas Red 1,2-Dihexadecanoyl-sn-Glycero-3-Phosphoethanolamine (Texas Red-DHPE; ThermoFisher Scientific, Waltham, MA) in molar ratio of 94.5/5/0.5 follow the same procedures in lipid vesicles preparation. After removal of excess lipids, Neutravidin™ (NA) was conjugated to the b-PE in the SLB via NA-biotin recognition, followed by conjugation of biotinylated EpAb4-1 (b-EpAb4-1) to the NA to complete the surface formation.

### Cell Line Used and Cell Culture

The human colorectal cancer cell line HCT116 and pancreatic ductal adenocarcinoma cell line AsPC1 were originally purchased from Bioresource Collection and Research Center (BCRC, Taiwan) and used as EpCAM positive cell line. The cells were maintained in Dulbecco’s Modified Eagle Medium (DMEM for HCT116; Gibco, Invitrogen Corporation, Carlsbad, CA) or RPMI-1640 (for AsPC1; Gibco) with 1% antibiotic-antimycotic (ThermoFisher Scientific) and 10% fetal bovine serum (FBS; Gibco) under 5% CO_2_ humidified atmosphere. The HCT116 cancer cells were pre-stained with CellTracker Green CMFDA dye (Life Technologies, Carlsbad, CA) or ectopic expression of red fluorescence protein (RFP) before spiking experiments. The sphere culture medium (SPH medium) was formulated as 10 ng/mL epidermal growth factor (EGF, Gibco), 10 ng/mL fibroblast growth factor (FGF; BD Biosciences, San Jose, CA), 10 ng/mL insulin (Sigma Aldrich, Saint Louis, MO), and B27 (Gibco) in serum-free DMEM medium. The eluted viable cells were then cultured either under complete DMEM medium or SPH medium with normal attachment culture dish (Corning, Corning, NY) or suspension culture by using an ultra-low attachment plate (Corning). Live/Dead assay (Life Technologies) was performed following the manufacturer’s instructions. The HS68 skin-derived non-tumorigenic human fibroblast cell line was maintained in low-glucose DMEM medium (Gibco) with 1% antibiotics and 10% FBS. For the cell lines used in this manuscript, the HCT116 and AsPC1 were originally purchased from Bioresource Collection and Research Center (BCRC, Taiwan).

### Capture Efficiency Test of the CMx Platform

The capture efficiency of the CMx platform was characterized using human colorectal cancer cell line HCT116. The HCT116 single cells pre-stained with CellTracker Green CMFDA (Life Technologies) were spiked into glass-bottomed wells (Diameter: 6 mm, Height: 5 mm) and 10 min was allowed for the cells to settle down. The well bottoms were imaged before and after cells were transferred to the whole blood collected from healthy individuals by fluorescence microscopy (Leica AF 6000 Advanced Fluorescence Imaging system) to ensure accurate counts. The actual number of spiked cells was defined as (cell number in well before transfer) minus (cell number remaining in well after transfer). After proper, gentle mixing, the cell-blood mixture was flowed through the functionalized microchannel under the flow rate of 1.5 mL/h. Afterward, the microchannel was washed with 0.5 mL of phosphate-buffered solution (PBS) under 4 mL/h flow rate and stained with 0.3 mL of Hoechst solution (1 μg/mL in PBS) for nuclei stain. The channel was photographed under fluorescent microscope (Leica DM16000B) to enumerate the total number of cells captured in the channel. Cancer cells capturing performance defined as the ratio of number of HCT116 cells bound on the chip to the number of cells being sent to the chip. For the test of capture and release efficiency of the cluster cells, incomplete digested HCT116 cells were selected by micromanipulator under microscope as previously described [6] and were directly spiked into the CMx platform. For both cell line and clinical samples validation, CTCs and CTMs were captured under same experimental conditions with same injection flow rate (1.5 mL/h) and PBS purification rate (4 mL/h).

### Immunofluorescence Staining

The captured cells, released from the chip onto the filter membrane, were fixed with 4% paraformaldehyde, permeabilized with 0.1% triton X-100 in 1x PBS, and blocked with bovine serum albumin (Millipore, Bedford, MA). Subsequently, cells were stained with rabbit anti-human cytokeratin 20 (CK20; Abcam, Cambridge, UK) and rat anti-human CD45 (Abcam) overnight at 4°C and followed by PBS wash. After PBS washing, cells were incubated with the FITC conjugated goat anti-rat IgG antibody (Abcam) and the Alexa Fluor^®^ 568 anti-rabbit IgG antibody (Life Technologies) for 1h at room temp, the excess antibodies were then removed by PBS washing and photographed by fluorescence microscope (Leica DM16000B). For immunostaining of fibroblast cells, α-smooth muscle actin (α-SMA, DAKO, Denmark) and fibroblast growth factor receptor (FGFR, Abcam) were used as fibroblast markers.

### Ethics Statement and Clinical Samples Collection

The peripheral blood samples were obtained from 54 CRC patients with stages I (n = 11), II (n = 13), III (n = 12) and IV (n = 18) with no prior cancer therapy and colon disease-free donors (n = 9) as negative controls were included in a double blinded, prospective study (31 female and 32 male). Total 2 mL of whole blood sample were collected by ethylene-diamine-tetra-acetic acid (EDTA) vacutainer tubes (BD Biosciences) from each patient and was used for both CTC and CTM capture on our platform for further analysis. The CRC patients were accepted in this study having no prior cancer treatments, receiving blood draw prior to the surgery on the same day or a day before the surgery. The healthy donors were confirmed by colonoscopy examination with no colon disease and received blood draw before the procedure. Average ages of patients and healthy donors are 62 and 47 years old, respectively. The patients were recruited from June to October 2012 in Chang Gung Memorial Hospital, Linkou, Taiwan. All samples were processed in Academia Sinica, Taipei, Taiwan. The clinical status and CTC results were double-blinded. The study protocol including experimental design and performance of this study were clearly described and was reviewed and approved by the Institutional Review Boards of Academia Sinica (AS·IRBOI-12040) and Chang Gung Memorial Hospital (100-1023B). The written informed consent was obtained from all patients including healthy volunteer and CRC patients before the study.

### Molecular Analysis

Total 7 mutation hotspots of KRAS (G12V, G12D), p53 (R248W, R175H, R248Q), and APC (E1309fs*4, 1556fs*3) mutation status were detected by commercially available TaqMan® mutation detection assay (Life Technologies) following the manufacturer’s instruction. In brief, the assay uses competitive allele-specific TaqMan PCR (castPCR technology). Each wild-type or mutant allele assay was composed of a modified or unmodified allele-specific forward primer, locus-specific TaqMan® probe, locus specific reverse primer, and allele-specific MGB blocker. Each test sample was run with a mutant allele assay(s) and the corresponding gene reference assay. Results were analyzed with the Seq Detection System version 2.3 to generate the values of CT_target_ and CT_reference_. The detected ΔCT cutoff value was used to determine the limit of the percent mutation in a sample that the mutant allele assay can detect. The conversion formula between % and ΔCT is 2^-(ΔCT)^ × 100%. The mutant allele assay sensitivity was 0.1%. Therefore, the value of ΔCT ≤ 9.96 is considered as positive and the value of ΔCT > 9.96 as negative.

### Statistical Analysis

Capture efficiency was reported as mean ± standard deviation. The group means were compared by an independent t-test. Differences were considered significant at the 95% confidence level (*p* < 0.05).

## Results

### Strategy of CTCs and CTMs Capture, Purification, and Release

**Fig 1:**
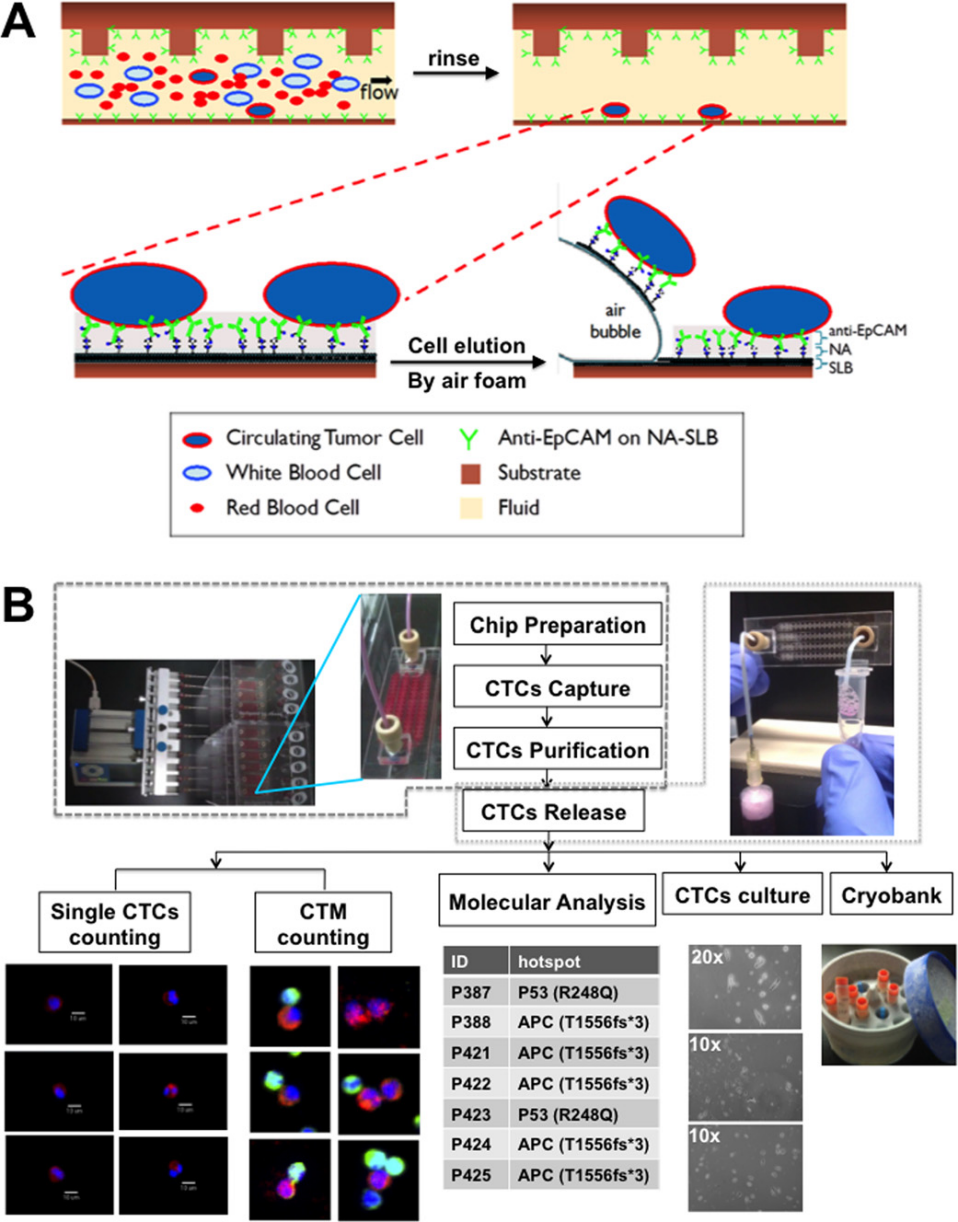
Overview of the CMx platform. (A) Strategies to capture, purify and release CTCs of the CMx platform. In the top row, blood flows through a microfluidic channel coated with anti-EpCAM conjugated to NeutrAvidin which is adhered to a SLB layer on substrate. Selective binding of CTCs to anti-EpCAM is reinforce by the antibody clustering effects through the mobility of the fluidic SLB layer, while other blood cells are easily flush away from the fluidic surface. The bottom row showed the release process, in which introduced air bubbles disrupt the weakest links between substrate and the SLB layer allowing elute intact CTCs for the collection outside of microfluidic chip. (B) Overview of CMx platform and summary of the CMx platform workflow. About 2 mL of the whole blood samples obtained freshly from the CRC patients were loaded equally into each CTC capturing devices. All chips went through cell capture, purification, and released for various downstream applications, including immunofluorescence staining, cell counting, molecular analysis, *ex vivo* cell culture and/or cryobanking.

The capture, purification, and release strategy takes advantage of SLBs to incorporate native-state proteins [46–53] and act as an interface between the inner wall of the microfluidic chip and antibody. The biomimetic SLBs inherently create a lubricating layer rejecting blood components (“non-fouling”) and assist the antibody’s mobility to form an excellent cell capture platform. In this work, a layer of antibody for epithelial cell adhesion molecules (anti-EpCAM), clone EpAb4-1, is conjugated to the SLBs for selective CTCs capture [46]. Previous studies have shown that protein-conjugated SLBs can create an excellent cell sorting and culture platform; moreover, the lateral fluidity of SLBs enables the clustering of proteins thus enhancing the binding affinity and specificity to the target cells [47,49]. Additionally, the zwitterionic nature of the lipid molecules in the SLB further minimizes non-specific protein and/or cell adsorption [48,49,54–56], thereby reducing fouling of the surface by peripheral blood. We incorporated the anti-EpCAM-SLB surface into a microfluidic chip with etched patterns designed to enhance chaotic mixing [57]. By controlling the fluid flow parameters, we first capture CTCs onto the surface and then increase the flow rate of buffer to diminish nonspecific adsorbed blood cells without displacing any of the bound CTCs (Fig 1A). Finally, purified CTCs are eluted from the chip via disrupting the SLB assembly, not the cell membrane or protein binding sites, by a gentle sweep of air foam. This combined strategy is able to accomplish the capture and purification of CTCs, subsequently allowing them to be released gently for further downstream molecular analysis or cultivation. The overview and the setting of the CMx plarform were shown in Fig 1B.

### Efficiency of Target Cell Binding and Capture

**Fig 2:**
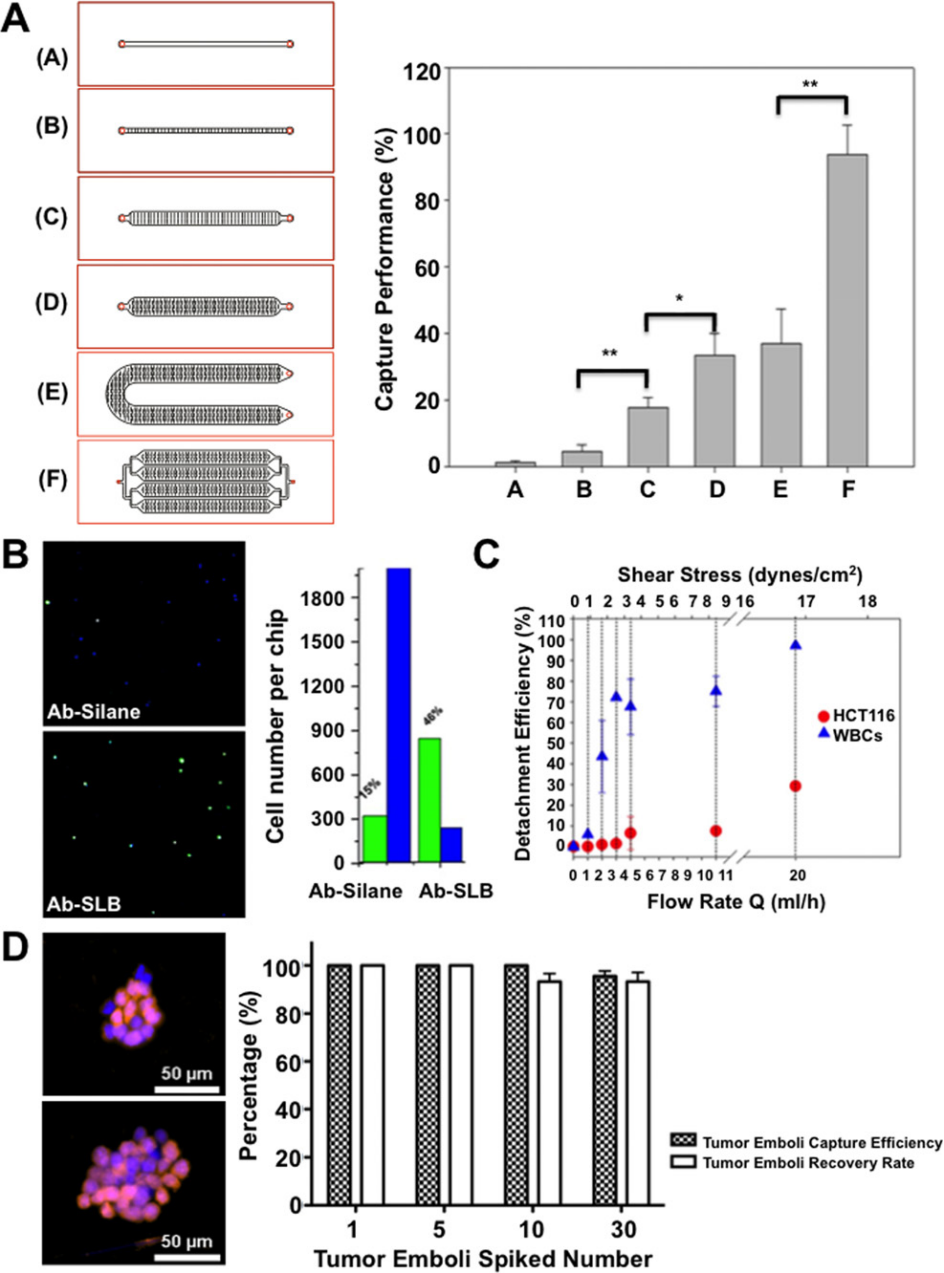
Capture performance and purification by Ab-SLB coated microfluidics. (A) The geometry and patterns of 6 different microfluidic channel designs (left) and the capture efficiency of these microfluidic platforms (right) as defined by dividing captured cells over total spiked cells. (B) The cropped fluorescent images (5.5 mm x 5.5mm) inside the flow channel and the enumeration of HCT116 (green) and WBCs (blue) on Ab-SLB or Ab-silane coated Type E chips. (C) Cell detachment efficiency (%, Y-Axis) vs. flow rates (ml/h, lower X-axis) and the corresponding shear stress (upper X-axis). Flow rates are generally maintained below 4 ml/h to avoid any potential loss of captured CTCs. (D) Highly CTM capture and recovery efficiency of the Ab-SLB coated chip. The capture efficiency and recovery rate of HCT116-RFP generated tumor microemboli were showed in right panel. The released HCT116-RFP CTC clusters with DAPI staining were showed in left panel. *: *p* < 0.05; **: *p* < 0.01.

The inner walls of the microfluidic channels were modified by lipid vesicles consisting of POPC and b-PE and conjugated of b-EpAb4-1 via NA-biotin recognition [46]. A series of microfluidic chips with different flow paths, dimensions, and microstructures were followed with the same surface modification (Fig 2A). Human colorectal cancer cell line HCT116 pre-stained with CellTracker Green CMFDA were then spiked into 1 mL whole blood collected from healthy individuals in EDTA tubes for target cell capture in a functionalized microchannel and followed with PBS purification and Hoechst nuclear staining. The total amount of cancer cells captured by the chip were then imaged and enumerated under a fluorescence microscope. The double-positive cells with both CellTracker Green and nuclear stain were identified as cancer cells. The single positive nuclear stain-only cells were identified as nonspecifically bound white blood cells (WBCs). The capture performance was defined as the ratio of number of HCT116 cells bound on the chip to the total number of cells spiked into the chip [21].

Fig 2A illustrates the geometry and patterns of 6 different microfluidic channel designs (Types A to F). Type A represents linear shape micro-channel without micro-patterns, Type B represents linear shape micro-channel with linearly arranged micro-patterns, Type C represents linear shape micro-channel with double-row linearly arranged micro-patterns, Type D represents linear shape micro-channel with alternate permutation linear micro-patterns, Type E represents U-shape micro-channel with alternate permutation linear micro-patterns, and Type F represents four-channel microfluidic with alternate permutation linear micro-patterns. Relative works were officially granted in September 2014 by US Patent and Trademark Office (US 20140255976 A1). The straight channels with simple patterns under same linear velocity represent very low capture performance due to the limited retention time and lack of mixing flow pattern: The capture performance of Type A chip = 1.2±0.6% and Type B chip = 4.5±2.2%. When the number of micro patterns increases to create efficient mixing, capture efficiencies increase substantially: Type C chip = 17.9±3.0% and Type D chip = 33.5±6.6%. Further doubling the channel length only slightly increased the efficiency to 37.0±10.5% (Type E), while 4 parallel channels further improved the capture efficiency to 93.7±8.9% (Type F). The spiking cell numbers were further lowered to the range of ~5 to ~100 per 2 mL blood to confirm the linearity of capture performance in Type F chip (S1A Fig).

### Non-fouling Property of the SLB Coated Surface and Target Cell Purification

To further evaluate the surface effect, a Type E chip was coated with conventional anti-EpCAM tethered silane (Ab-Silane) or anti-EpCAM conjugated SLB (Ab-SLB). Fig 2B showed that the capture performance of HCT116 is about 15% on the Ab-Silane coated chip, with over 2000 WBCs being non-specifically bound on the chip. In comparison, the Ab-SLB coated chip significantly enhanced capture efficiency and reduced non-specific WBC on the chip.

The purity of the captured cells can be further improved by simply increasing the flow rate of the PBS buffer flush in an antibody-SLB coated chip. As the flow rate of PBS increased, the percentage of retained WBCs decreased significantly. In our study, the elevated shear stress to remove 55~80% of WBCs while retaining more than 90% HCT116 requires ~4–8 dynes/cm^2^ (corresponding to flow rates of ~5–10 mL/h, Fig 2C). The shear stress required to purify target cells is substantially lower than what was reported in previous cell detachment studies based on conventional antibody silanized surfaces and is considered low enough to have minimal or no impact on cell viability or protein expression [40,58]. On the contrary, HCT116 remained bound even at 50 mL/h due to the strong antibody-antigen interaction (S1 Movie). For the clinical samples, we chose a smaller flow rate of 4 mL/h to avoid any potential loss of CTCs.

### Release of Captured Viable Target Cells

**Fig 3:**
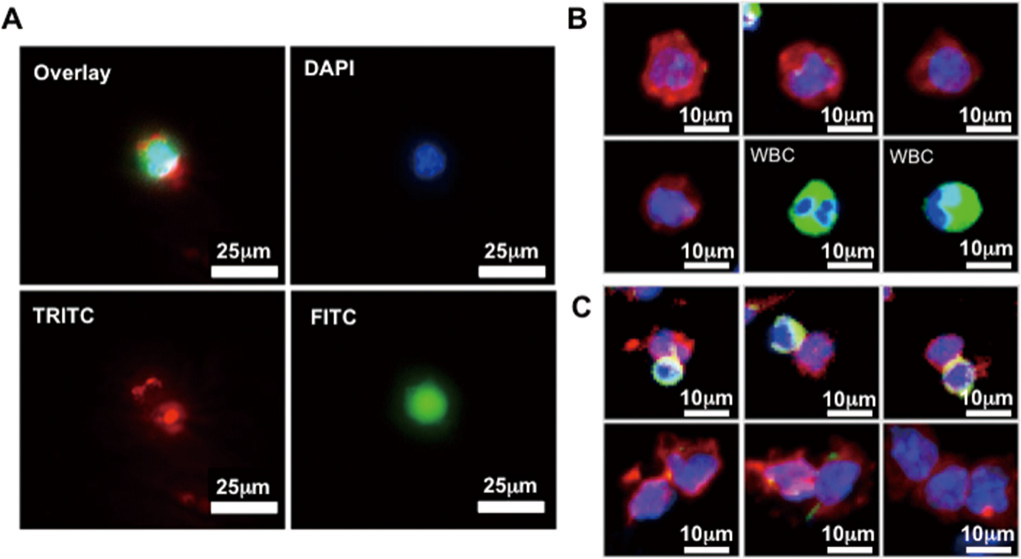
Release of captured cells by air foams from CMx platform. (A) The florescent images of the HCT116 cancer cell released from the Texas Red-SLB coated chip. The cell was wrapped with Texas Red-SLB lipid molecules around the membrane (blue: DAPI; green: pre-stained CellTracker Green CMFDA; red: Texas Red conjugated lipid molecule). (B and C) Overlaid fluorescent images of the released single CTC and CTM for cell characterization. Eluted cells from clinical samples were categorized as CTC by size, morphology and immunostainning (DAPI+/CK20+/CD45-). WBCs were identified by DAPI+/CK20-/CD45+ immunostaining (blue: DAPI; green: CD45; red: CK20).

Releasing viable CTCs from the microfluidic chips is a critical step enabling convenient cell preservation, cell culture, and downstream molecular analysis. Previous attempts using enzymatic cleavage [34,36] or mechanical forces [40,58] to detach cells have shown either partial release or inevitable cell death, attributed by the breakage of antibody-antigen linkages or membrane rupture. The SLB is a lipid molecular assembly at the water-solid interface stabilized by inward hydrophobic-hydrophobic interactions of long hydrocarbon chains of lipid molecules and outward hydrophilic head groups interacting with water or hydrophilic silicon oxide surfaces (i.e., glass). The SLB assembly could be easily disintegrated by introduce a hydrophobic component as simple as air bubbles. We offered a strategy to gently release adhesive CTCs without disrupting the antibody-antigen bonds by injecting continuous air foam to the microfluidics (S2 Movie). The foamy solution was created by a mixture of air with cell culture medium and gently vortexed for foam creation. The average release efficiency using 250 μl foamy solution was 99.7% in 3 repeats (S1B Fig). In order to validate the cell integrity released from the microfluidic chips using air foam, HCT116 cells were pre-stained with CellTracker Green CMFDA and the Texas Red-SLB were used for surface coating indication. After released by air foam, the cells were stained with DAPI nuclear stain and photographed under fluorescence microscope. Fig 3A showed that the eluted cell was wrapped by lipids, indicating cell release through peeling off SLB by air. Live/Dead assay was performed immediately after cell elution; results showed 86% of eluted cells remain viable as compared to 0% viability from the conventionally coated anti-EpCAM silanized chip (S2 Fig). By introducing hydrophobic air foams, gentle release of the captured CTCs could be accomplished without damaging the cells. As a result, the non-fouling SLB surface preserved intact cell morphology and bound viable cells for further investigation.

### Capture and Release of CTMs

In order to validate the capability of Ab-SLB coated microfluidic chip for CTMs capture and release, the RFP ectopically expressed HCT116 were used. The *ex vivo* tumor emboli were simulated using incomplete digestion by trypsin and selected by micromanipulator under microscope as previously reported [6]. Diverse numbers of selected HCT116-RFP emboli were spiked into the microfluidic chips for further capture and release efficiency testing. The released emboli were then imaged under fluorescent microscope after DAPI nuclear stain (Fig 2D, left panel). The capture efficiency of 1, 5, 10, 30 emboli spiked test were 100%, 100%, 100%, 96%, respectively (Fig 2D, right panel). The recovery rate of 1, 5, 10, 30 emboli spiked test were 100%, 100%, 93%, 93%, respectively. All results were obtained in triplicate with the same protocol. The results indicate that both CTCs and CTMs were able to capture in our platform with high efficiency and feasible for further clinical testing.

### Detection of CTCs from CRC Patients of All Stages

An important advantage of easy cell release from a microfluidic device is that cells can be collected and stained on a small planar substrate. Instead of the in-chip inspection within the 3-dimensional complex microfluidic channels, imaging CTCs on a much smaller area (~10 mm in diameter) enables easy relocation of the cells of interest, and significantly reduces imaging and inspection time from 6h to <0.5h (S3 Fig). Furthermore, the cells released by gentle air foam retained intact cell morphology, greatly alleviate the imaging errors and boosting the accuracy. As a result, high throughput of clinical sample analysis is probable.

We tested the Type F platform with peripheral human blood samples from 54 CRC patients and 9 colon disease-free donors. Assay procedures were mostly identical to the spiked experiments: After CTC capture and purification, air foam sweep was used to release captured CTCs onto a small membrane with 2 μm pore size to drain the excessive staining solutions where immunofluorescence staining was performed to identify and enumerate the released cells including single CTCs and CTMs (Fig 3B and 3C). The monoclonal antibody against CK20 was used as a positive staining marker as it is more colorectal-specific than pan-CKs [59]. The anti-CD45 antibody was used for the staining of WBCs. The patient-derived CTCs were defined as CK20+/CD45-/DAPI+ cells, and the WBCs were defined as CK20-/CD45+/DAPI+ cells.

**Fig 4:**
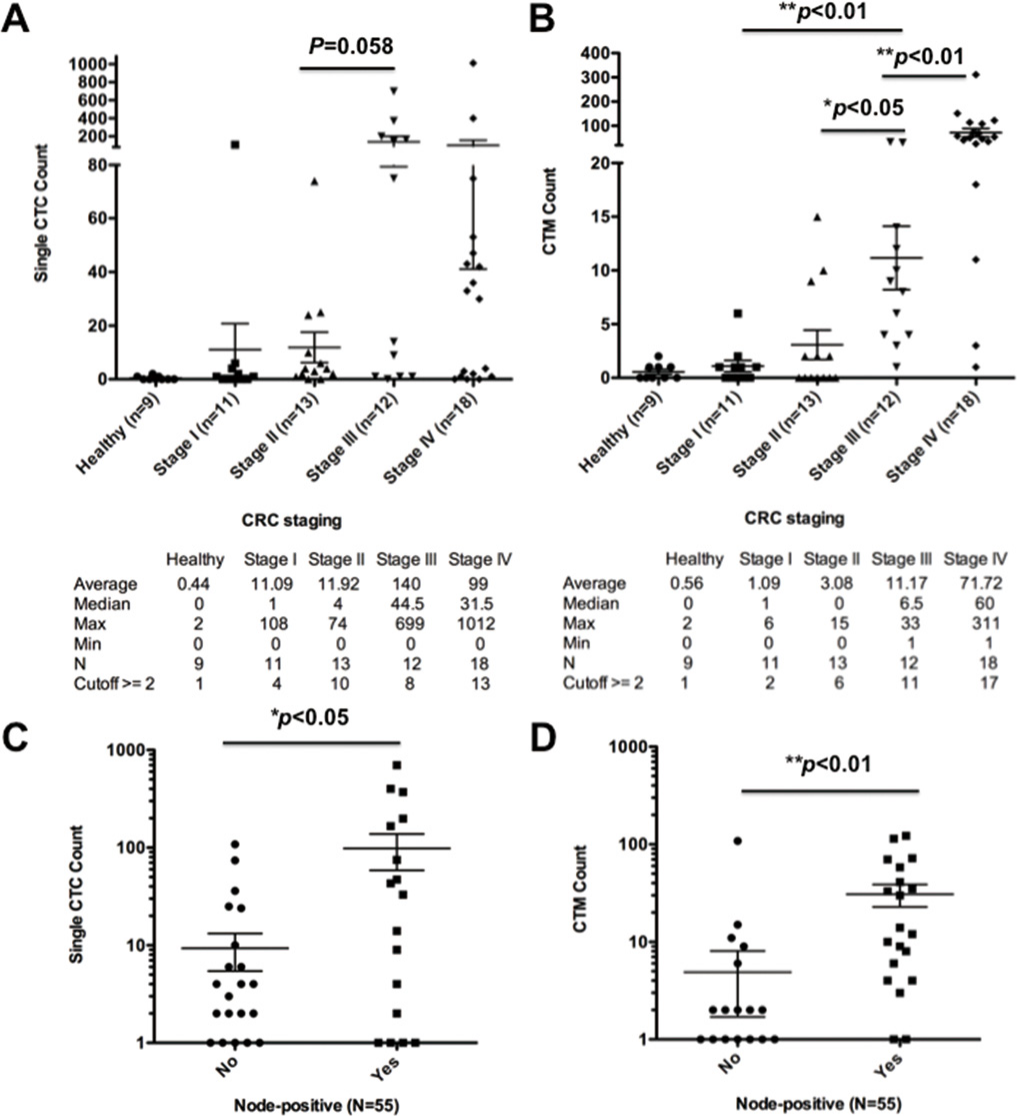
Enumeration and correlation of single CTCs and cluster CTCs with disease progression. (A) Correlation and statistical analysis of single CTC enumeration and clinical stage of CRC patients and healthy donors. (B) Correlation and statistical analysis of CTM enumeration and clinical stage of CRC patients and healthy donors. Correlation between (C) CTCs and (D) CTM and lymphnode metastasis of CRC patients were shown with mean±SEM. (* *p* < 0.05, ** *p* < 0.01)

The single CTC enumeration from the patients showed increase of CTCs followed with advance of CRC disease stages, and the CTCs found were relatively low but detectable in early stage CRC patients (Fig 4A). Among 9 healthy donors, CTCs were rarely found and ranged from 0 to 2. The median numbers of CTC cells in healthy, stage I, stage II, stage III, and stage IV were 0, 1, 4, 44.5, and 31.5, respectively. The mean numbers of CTCs were 0.4, 11.1, 11.9, 140, and 99, respectively from healthy to stage I-IV CRC patients. The CTC counts of stage IV CRC patients were broadly distributed, with the highest cell count exceeding 1000. The mean values and standard deviations of each clinical stage CRC patient are shown in Fig 4A. By using 2 CTCs as positive detection value, the sensitivity, specificity, and positive prediction rate of CMx platform in CRC cancer detection from healthy donors are 65%, 89%, and 97%, respectively.

### Significant Positive Correlation of CTMs with the Disease Progression in CRC Patients

A total of 54 CRC patients and 9 healthy donors were randomly selected for CTM enumeration from the same patient pool identified in single CTC enumeration. A significant correlation between CTM numbers and disease progression was demonstrated in CRC patients and healthy donors (Fig 4B). In comparison of single CTC and CTM count in the same group of CRC patients, CTM enumeration was able to distinguish the stage progression significantly between CRC patients (Fig 4A and 4B). Among 9 healthy donors, CTM were rarely found and ranged from 0 to 2. The median numbers of CTM in healthy, stage I, stage II, stage III, and stage IV were 0, 1, 0, 6.5, and 60, respectively. The mean numbers of CTM were 0.6, 1.1, 3.1, 11.2, and 71.7, respectively, from healthy to stage I-IV CRC patients. By using ≥2 CTM in 2 mL blood as positive selection criteria, the sensitivity, specificity, and positive prediction rate of the CTM to distinguish cancer patients from healthy samples were 67%, 89%, and 97%, respectively.

Particularly, the numbers of both single CTC and CTM were significantly higher in lymph node-positive CRC cancer patients than those CRC patients without lymphatic metastasis (Fig 4C and 4D). The medium numbers of CTC/CTM in lymph node-negative or positive patients were 1/0.5 (n = 34) and 9/12 (n = 21), respectively. The mean numbers of CTC/CTM in lymph node-negative or positive patients were 9.3/0.5 (n = 34) and 98.3/30.8 (n = 21), respectively.

### The CMx Platform Enables Viable CTCs Elution for Further *ex vivo* Cultivation

**Fig 5:**
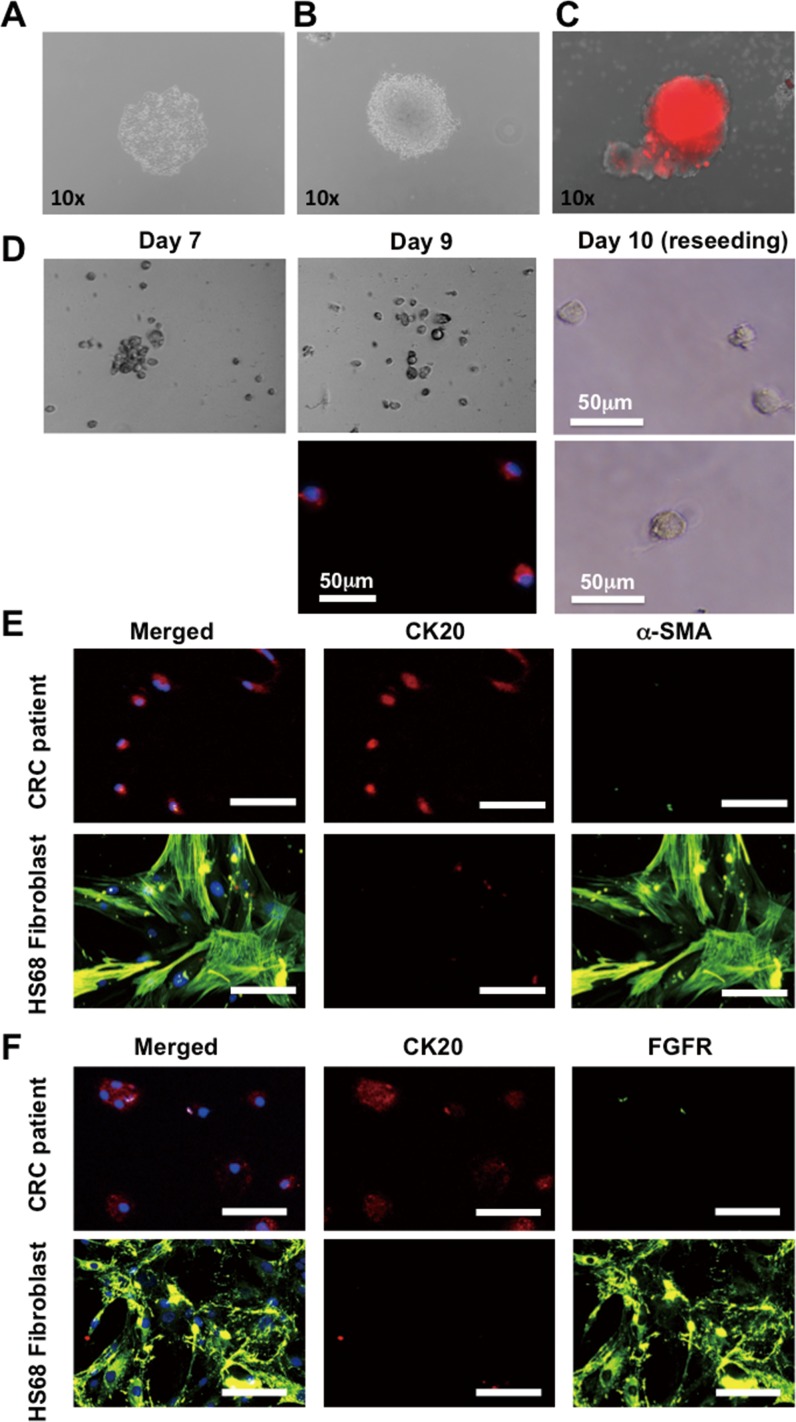
Cultivation of viable eluted CTCs. Capture and release of HCT116 cancer cell from CMx platform for further cultivation under (A) complete DMEM medium, (B) SPH culture medium, (C) suspension culture with SPH medium. (D) CTCs isolated from a stage III CRC patient. The cells gathered and attached to the bottom of culture plate at day 7 and became more spread out at day 9. Cells exhibited CK20+ (red) and nuclei+ (blue) with a size around 25μm. To prove these cells have a propensity to re-attach, they were treated with 0.1% trypsin and re-seeded at day 9. After 1 day, these cells re-attached to the substrate firmly (day 10). (E and F) Immunocytochemistry stain for the confirmation of colorectal origin of patient-derived CTC primary cultured cells. The skin-derived fibroblast cell line HS68 was used as negative staining control (blue: DAPI; red: CK20; green: α-SMA or FGFR).

To determine whether the isolated cells can be cultured *ex vivo*, air foam released HCT116 cancer cells were collected and seeded onto adequate tissue culture plates with selected medium. The released HCT116 were viable and able to be cultured in advance to form a single clone colony under complete DMEM medium (Fig 5A). In addition, with SPH medium, the released HCT116 were able to maintain stemness properties in formation of stem cell enrichment colony and sphere under both attachment and suspension culture condition (Fig 5B and 5C). To further evaluate the ability of the CMx platform for patient-derived primary CTC culture, cancer cells isolated from a stage III patient were released into 96-well attachment culture dish for further cultivation. The cells were gathered and attached to the bottom of the culture dish at day 7 and became more spread out at day 9 (Fig 5D). The immunofluorescence staining confirmed the double positive staining of both CK20 and DAPI nuclear stain (Fig 5D, lower middle panel). To prove the cells have a propensity to re-attach, the cells were detached with 0.1% trypsin and re-seeded onto 96-well plate at day 9. After 1 day, these cells (day 10) re-attached to the substrate firmly with an apparent cell size ~20 μm (Fig 5D, right panel). Although proliferation was not observed due to the challenging *ex vivo* growth environment, maintenance of CTC viability was guaranteed in our platform for at least two months. The origin of patient-derived CTCs released by the CMx platform was also confirmed by immunostaining using FGFR and α-SMA fibroblast markers and CK20 (Fig 5E and 5F). Positive CK20 and negative in fibroblast markers staining represent the epithelial origin of the cells obtained from the whole blood of CRC cancer patients. Whereas HS68 skin-derived human fibroblast cells represent α-SMA and FGFR instead of CK20, were used as fibroblast positive control.

### Hotspot Mutation Detection in Purified Patient-derived CTCs

For further characterize the patient-derived CTCs captured by CMx platform, 7 CRC cancer mutation hotspots were identified. The DNA was extracted directly from the eluted cells to perform castPCR for cancer mutation hotspots in p53, adenomatous polyposis coli (APC) and KRAS mutations (Table 1). The results of 3 healthy donors were all negative (ΔCt >9.96); Among 10 randomly selected CRC patients, 2 samples were positive (ΔCt <9.96) for TP53 R248Q mutation and 5 were positive for APC1556fs*3 mutation. The results confirmed the cancer origin of the captured CTCs with compact nuclear and intact genetic materials for further investigation and diagnosis application.

**Table 1:** Genetic mutation analyses of eluted CTCs. Hotspot analyses of randomly selected 3 healthy (H1~H3) and 10 colorectal cancer patient samples (P1~P10) by castPCR. The mutation loci detected include 3 loci for p53, 2 loci for APC, and 2 loci for KRAS. (Loci with ΔCt value < 9.96 indicate positive and were represented in bold format).

		TP53R248W		TP53R175H		TP53R248Q		APC1309fs*3		APC1556fs*3		KRASG12V		KRASG13D	
ID	CTC/2mL	Ct	ΔCt	Ct	ΔCt	Ct	ΔCt	Ct	ΔCt	Ct	ΔCt	Ct	ΔCt	Ct	ΔCt
H1	0	NVD	NA	NVD	NA	35.0894	11.257	NVD	NA	33.9754	10.2039	NVD	NA	NVD	NA
H2	0	NVD	NA	36.2758	12.3402	NVD	NA	NVD	NA	33.7421	10.7723	NVD	NA	NVD	NA
H3	0	39.2909	15.4595	34.6825	10.8511	NVD	NA	NVD	NA	33.6487	10.7724	NVD	NA	NVD	NA
P1	174	NVD	NA	38.8593	14.4028	33.831	**9.3745**	NVD	NA	37.9785	13.7704	NVD	NA	NVD	NA
P2	28	NVD	NA	NVD	NA	NVD	NA	NVD	NA	33.1786	**8.5154**	NVD	NA	NVD	NA
P3	26	NVD	NA	39.8006	15.3288	NVD	NA	NVD	NA	35.7989	11.1062	NVD	NA	NVD	NA
P4	12	NVD	NA	39.1121	15.078	37.81	13.7759	NVD	NA	32.861	**8.8555**	NVD	NA	NVD	NA
P5	6	NVD	NA	37.5928	13.5116	NVD	NA	NVD	NA	33.3171	**9.2162**	NVD	NA	NVD	NA
P6	2	NVD	NA	35.608	10.9916	34.1655	**9.5491**	NVD	NA	NVD	NA	NVD	NA	NVD	NA
P7	2	NVD	NA	38.4027	13.9292	NVD	NA	NVD	NA	34.4292	**9.6906**	NVD	NA	NVD	NA
P8	6	NVD	NA	39.4417	15.4701	NVD	NA	NVD	NA	32.0008	**8.5251**	NVD	NA	NVD	NA
P9	4	NVD	NA	38.6133	14.5613	39.4851	15.4331	37.0999	12.8839	36.1388	11.9228	NVD	NA	NVD	NA
P10	4	NVD	NA	NVD	NA	NVD	NA	NVD	NA	34.9676	10.9355	NVD	NA	NVD	NA

## Discussion

We have developed a microfluidic system for simple and effective capture, purification, release, and collecting of CTCs by using only 2 mL of peripheral whole blood directly from all stages of cancer patients without any further processing. The purified patient-derived intact and viable CTCs enable further investigation through direct *in vitro* cultivation or nucleic acid extraction for genetic research in advance. The strategy to retrieve sizable CTCs was inspired by the cell membrane through non-covalently bound SLB to promote dynamic clustering of lipid-tethered antibodies to antigens on CTCs and minimizing non-specific blood cell by its non-fouling nature. The lipid molecules in the SLB are stabilized based on hydrophilic/hydrophobic interactions, not by covalent bonding, as a result, the conjugated antibody molecules can easily migrate toward the target cells due to either active recruitment or molecular diffusion [53,60]. As a result, antibody-antigen clustering, cells are adhered more firmly on the surface than those on a conventional antibody immobilized surface with pre-fixed density. Conversely, when the antibody-functionalized lipids are concentrated underneath the target cells, the concentration of non-functionalized lipids away from the target cells increases, leading to greater resistance to non-specific binding. Reduced non-specific binding by protein and blood cells also reduces the steric hindrance created by their physical occupancy on the limited binding space, thus becoming more accessible for incoming CTCs. With the mobilized lipid coating surface, the Ab-SLB microfluidic chip thus enables gathering purified CTCs with high sensitivity and specificity.

The non-fouling property of the SLB surface provides advantage in eliminating non-specifically bound blood cells after exerting small force of PBS wash as we previously reported [46]. The shear stress required to remove WBCs from the SLB surface is much less than that from a conventional antibody coated surface, and is much lower than normally experienced by cells in physiological state such as that observed in human blood vessels (~15 dyne/cm^2^). Through hydrophobic air foam introduction, the CTCs captured by our platform were able to be release completely without damage. The purified intact and viable CTCs were feasible for further investigation including genetic material extraction, molecular analysis, and *in vitro* cultivation of patient-derived CTC cells.

The widely used Veridex CellSearch CTC enumeration system approved by the US FDA demonstrated that only 25% of stage IV CRC patients have ≥3 CTCs per 7.5 mL blood (defined as panCK+/CD45-/DAPI+), and the median CTC number of stage IV patients is zero [61]. Rather by using panCK as the detection marker in CRC, we use CK20 instead, as it has been a reliable marker to distinguish colonic adenocarcinoma, expressed virtually in all cases of CRC tumor [62,63]. Under the scenario for specific detection of CTCs in CRC, in our clinical study, CK20 has shown to be the prevailing target for both identification and detection of CTCs from the whole blood CRC clinical samples.

By using CK20+/CD45-/DAPI+ as selection criteria and ≥2 CTCs per 2 mL blood as a cutoff, our platform obtained 70% of sensitivity, 89% of specificity, and 96% of positive prediction rate for the metastatic stage III-IV CRC patients from healthy donors except one out of 9 healthy donors has CTC count to two. By using ≥2 CTM per 2 mL blood as a cutoff, the sensitivity, specificity, and positive prediction rate for metastatic stage III-IV CRC patients from healthy donors were 93%, 89%, and 97%, respectively. In consideration of both single CTC and CTM, combining single CTC and CTM as criteria (either ≥2 CTCs or ≥2 CTM) for late stage (III-IV) CRC patient validation from healthy donors, high overall sensitivity (93%), specificity (89%), and positive prediction rate (97%) were evaluated by our platform. In addition, for detection and diagnosis of early stage (I-II) CRC patients, our platform showed good overall sensitivity (67%), specificity (89%), and positive prediction rate (94%) from healthy donors. About 82% overall sensitivity, 89% overall specificity, and 98% overall positive prediction rate were obtained to distinguish patients with CRC from the healthy donors. The distribution of CTC and CTM counts of the validated CRC patients were show in S4 Fig. Reduced sample requirement and higher sensitivity of the CMx platform enable consistent and stable diagnosis for clinical usage.

The 5-year survival rate of localized, regional, or distant CRC in US are 40%, 36%, 20%, respectively [64]. The cancer patients may have had CTC in circulation and micro-metastasis undetected by the currently available diagnostic techniques prior to the surgery and result in the high recurrence and poor survival rate even after the presumably curative surgical resection. A platform that provides sensitive and reliable outcomes in CTC enumeration will offer a great potential for early cancer detection and real-time surveillance during disease treatment when disease is more curable. In this study, we showed that 36.4% (4/11), 76.9% (10/13), 66.6% (8/12), and 72.2% (13/18) of stage I, II, III, and IV CRC patients, respectively, had positive CTC counts exceeding the cutoff value of 2. The positive CTC incidence rates correspond to the recurrence rates with disease progression. CTCs are the cells that successfully invade the circulation system and survive at the time of blood draw. Though CTCs are the precursors and do not necessarily lead to the extravasation and eventual colonization, those early staged cancer patients in our study with high CTC counts were considered at high risk for potential metastasis and are subject to follow-up.

CTC clusters, namely, aggregates of more than 2 CTCs, have been identified in peripheral blood as circulating microemboli of various cancer patients including colorectal, prostate, breast, and small cell lung cancers a decade ago [1,5,6,65]. Not until the CTCs capturing devices had been developed could the clinical importance of CTC clusters be then shown in both breast and prostate cancers [6]. The existing CTC clusters in circulation have also been reported to correlate with poor prognosis and distant metastasis of the cancers, which may be the result of enhancing E-selecting mediated rolling adhesion in vasculature [6,66]. Since the interaction between immune cells and cancer cells are required for immune response, mutations in cancer cells may further take advantage during immune contact and facilitate its metastatic progression and survival in circulation [67]. The interaction between CTCs, WBCs, and endothelial cells through adhesion molecules or cell surface ligand-receptors conjugation will further provide survival advantage to the cancer cell traveling through the blood stream [68]. The presence of both CTC clusters and CTC-WBC company clusters had already been found in patients with colorectal, breast, prostate, and lung cancer [21,65,69,70]. In addition, it had been reported that infiltrating WBCs were capable of fusion with tumor cells and facilitate its intravasation into circulation, subsequently triggering invasion and metastasis of the tumor [71,72]. The CTC-WBC company clusters may thus play an important role during cancer progression. On the other hand, purification of CTCs by negative selection using CD45-conjugated magnetic beads may lose the information of the CTC-WBC company clusters that comprise the main population in the CTC microemboli we observed (data not shown). However, the clinical significance of CTC-WBC company clusters has not been clearly validated. Enumeration of CTMs including both CTC-only clusters and CTC-WBC company clusters may improve the clinical diagnosis of the cancer patients. In our case, enumerating CTMs provide solid evidence in distinguishing different stages of CRC patients. The results showed that about 18.2%, 46.2%, 91.7%, and 94.4% of stages I, II, III, and IV CRC patients, respectively, had positive CTM counts exceeding the cutoff value of 2, demonstrating that the CTM highly correlated with disease progression and possibly a better factor for the diagnosis and prognosis prediction of CRC patients.

## Conclusion

We have demonstrated a strategy for simple capture, purification, and release of CTCs that uses an antibody conjugated non-fouling SLB film. Fig 1B shows a simple workflow of our economic system for capture, purification, and release in a continuous and parallel process. 8mL of whole blood is sufficient for all purposes including the enumeration of CTCs and CTM, cell culture, cryobank storage, and molecular analysis (Table 1). A typical 1mL blood sample contains ~6.6x10^6^ nucleated WBCs, and the SLB coated microfluidic system eliminates 99.99% on average. Currently, about 500–2000 WBCs were eluted with the CTCs mostly due to dead volume in the fluidic device and tubing. Nevertheless, this purity level of CTCs is sufficient to facilitate molecular analyses for oncogene identification (0.1% purity for cast PCR) and possibly offer benefits of early intervention using appropriately selected therapy. In conclusion, our platform offers the complete solution for CTC research for imaging, molecular analysis, cell culture, and preservation simultaneously, and requires only a minimum volume of whole blood sample. Development of Ab-SLB coated microfluidic devices opens up the opportunity for clinical diagnosis and will benefit for further investigation in the field of cancer metastasis.

## Supporting Information

S1 Fig Capture and release efficiency of the spiked cancer cell lines. (DOCX)

S2 Fig Fluorescent images of HCT116 viability immediately release after anti-EpCAM-SLB or anti-EpCAM silanized chips capture. (DOCX)

S3 Fig Collection of eluted cells onto a planar porous membrane for convenient immunofluorescent staining and enumeration. (DOCX)

S4 Fig CRC patient distribution of both single CTC and CTM counts by clinical stage. (DOCX)

S1 Movie Real time monitoring of purifying non-specifically bound WBCs using flow. (AVI)

S2 Movie Release of captured CTCs by air bubbles. (AVI)
