# Supplementary material for: Sensitive and Specific Biomimetic Lipid Coated Microfluidics to Isolate Viable Circulating Tumor Cells and Microemboli for Cancer Detection
Source: PLoS One. 2016 Mar 3;11(3):e0149633. doi: 10.1371/journal.pone.0149633 (PMC4777486; doi:10.1371/journal.pone.0149633)
Supplement: S1 Fig — (DOCX) [file pone.0149633.s001.docx]

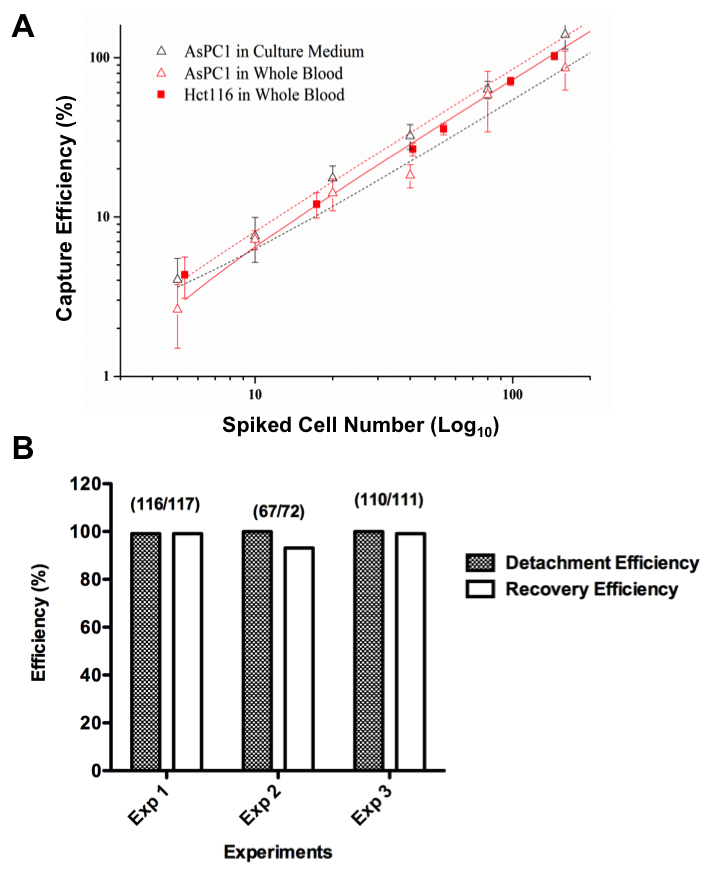


**S1 Figure**. **Capture and release efficiency of the spiked cancer cell lines**.

**(A)** The capture performance of Type F chip using both HCT116 and AsPC1 in culture medium and whole blood (2 mL). The spiked cell numbers are ranged from ~5 to ~100/ 2 mL. The linear regression of each cell line: HCT116 in blood: y = 0.7359x - 0.90844, R2 = 0.99723. Pancreatic Cancer AsPC1 in medium: y = 0.85148x - 0.43378, R2 = 0.99626. AsPC1 in blood: y = 0.53265x + 0.9605, R2 = 0.93984. Each point is averaged based on three repeat experiments. **(B)** HCT116 single cell release efficiency of the CMx platform. Note: The detachment efficiency is estimated by the number of cells in chip after air foam sweep to the original number of cells captured by the chip. The recovery efficiency is estimated by the number of cells actually acquired on the member to the number of original captured cells.
