# Supplementary material for: Sensitive and Specific Biomimetic Lipid Coated Microfluidics to Isolate Viable Circulating Tumor Cells and Microemboli for Cancer Detection
Source: PLoS One. 2016 Mar 3;11(3):e0149633. doi: 10.1371/journal.pone.0149633 (PMC4777486; doi:10.1371/journal.pone.0149633)
Supplement: S2 Fig — (DOCX) [file pone.0149633.s002.docx]

**
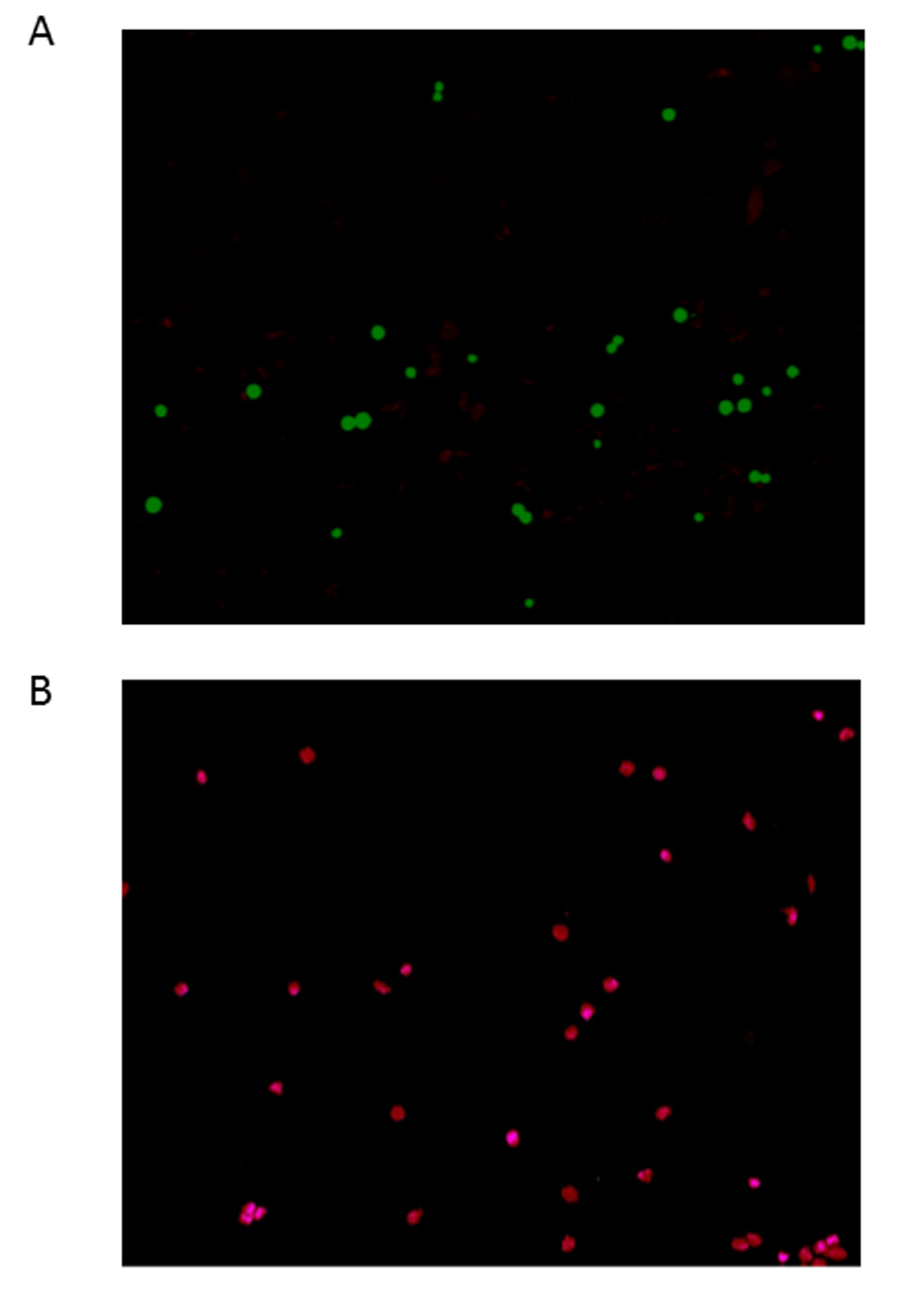
**

**S2 Figure**. **Fluorescent images of HCT116 viability immediately release after anti-EpCAM-SLB or anti-EpCAM silanized chips capture.**

Fluorescent images showed the viability of HCT116 cells stained by LIVE/DEAD^®^ reagent, immediately after (A) cells were released from the anti-EpCAM-SLB, and (B) anti-EpCAM silanized chips. Membrane-permeated calcein AM is cleaved by esterases in live cells to yield cytoplasmic green fluorescence, and membrane-impermeant ethidium homodimer-1 labels nucleic acids of membrane-compromised cells with red fluorescence. The estimated viability is (A) 86%, and (B) 0%. The image photographed under 10x object lens.
