# Supplementary material for: Sensitive and Specific Biomimetic Lipid Coated Microfluidics to Isolate Viable Circulating Tumor Cells and Microemboli for Cancer Detection
Source: PLoS One. 2016 Mar 3;11(3):e0149633. doi: 10.1371/journal.pone.0149633 (PMC4777486; doi:10.1371/journal.pone.0149633)
Supplement: S3 Fig — (DOCX) [file pone.0149633.s003.docx]

**
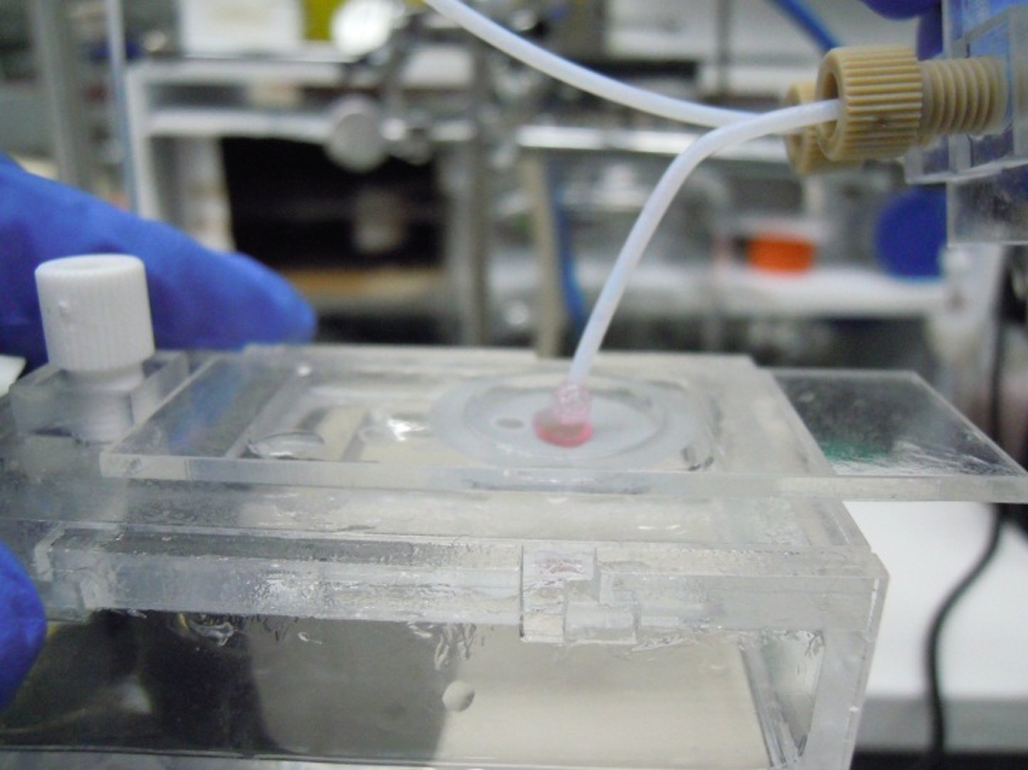
**

**S3 Figure**. **Collection of eluted cells onto a planar porous membrane for convenient immunofluorescent staining and enumeration**.

Eluted cells can be collected onto a planar porous membrane (shown diameter 10 mm) for convenient immunofluorescent staining and enumeration.
