# Supplementary material for: Sensitive and Specific Biomimetic Lipid Coated Microfluidics to Isolate Viable Circulating Tumor Cells and Microemboli for Cancer Detection
Source: PLoS One. 2016 Mar 3;11(3):e0149633. doi: 10.1371/journal.pone.0149633 (PMC4777486; doi:10.1371/journal.pone.0149633)
Supplement: S4 Fig — (DOCX) [file pone.0149633.s004.docx]

**
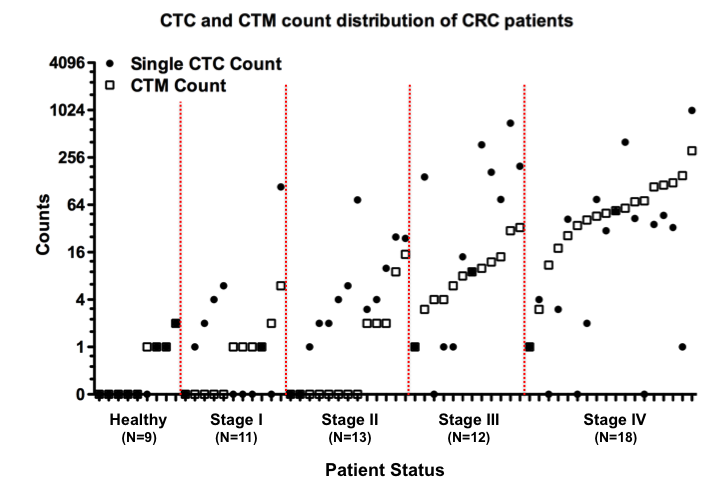
**

**S4 Figure**. **CRC patient distribution of both single CTC and CTM counts by clinical stage**.

Single CTC and CTM counts per 2 mL of peripheral blood for total 83 patients from healthy to stages I-IV CRC patients. CTC counts were presented in log 2 scale.
